# Parasite‐associated mortality in a long‐lived mammal: Variation with host age, sex, and reproduction

**DOI:** 10.1002/ece3.3559

**Published:** 2017-11-12

**Authors:** Carly L. Lynsdale, Hannah S. Mumby, Adam D. Hayward, Khyne U. Mar, Virpi Lummaa

**Affiliations:** ^1^ Department of Animal and Plant Sciences University of Sheffield Sheffield UK; ^2^ Department of Zoology University of Cambridge Cambridge UK; ^3^ Department of Environmental Sciences Applied Behavioural Ecology and Ecosystem Research Unit University of South Africa Johannesburg South Africa; ^4^ Department of Biological and Environmental Sciences University of Stirling Stirling UK; ^5^ Department of Biology University of Turku Turku Finland

**Keywords:** individual variation, infectivity, life history, parasitism, trade‐off, vertebrate

## Abstract

Parasites can cause severe host morbidity and threaten survival. As parasites are generally aggregated within certain host demographics, they are likely to affect a small proportion of the entire population, with specific hosts being at particular risk. However, little is known as to whether increased host mortality from parasitic causes is experienced by specific host demographics. Outside of theoretical studies, there is a paucity of literature concerning dynamics of parasite‐associated host mortality. Empirical evidence mainly focuses on short‐lived hosts or model systems, with data lacking from long‐lived wild or semi‐wild vertebrate populations. We investigated parasite‐associated mortality utilizing a multigenerational database of mortality, health, and reproductive data for over 4,000 semi‐captive timber elephants (*Elephas maximus*), with known causes of death for mortality events. We determined variation in mortality according to a number of host traits that are commonly associated with variation in parasitism within mammals: age, sex, and reproductive investment in females. We found that potentially parasite‐associated mortality varied significantly across elephant ages, with individuals at extremes of lifespan (young and old) at highest risk. Mortality probability was significantly higher for males across all ages. Female reproducers experienced a lower probability of potentially parasite‐associated mortality than females who did not reproduce at any investigated time frame. Our results demonstrate increased potentially parasite‐associated mortality within particular demographic groups. These groups (males, juveniles, elderly adults) have been identified in other studies as susceptible to parasitism, stressing the need for further work investigating links between infection and mortality. Furthermore, we show variation between reproductive and non‐reproductive females, with mothers being less at risk of potentially parasite mortality than nonreproducers.

## 1 INTRODUCTION

Parasites have detrimental effects on host health and fitness (Soares, Gozzelino, & Weis, 2014), driving infectious disease (Simpson, Johnson, & Carver, 2016) and posing major conservation threats, particularly to endangered or isolated host species (Pedersen, Jones, Nunn, & Altizer, 2007; Wikelski, Foufopoulos, Vargas, & Snell, 2004). At the population level, high infection intensity can disrupt group dynamics and substantially limit population growth and size (Albon et al., 2002; Eira, Vingada, Torres, & Miquel, 2006; Watson, 2013), sometimes accelerating population crashes or elevating extinction risk (Brzeski et al., 2015; Gulland, 1992; Johnson et al., 2012). At the individual level, heavy burdens can be highly pathological, increasing risk of infectious disease and secondary illnesses (Beldomenico et al., 2008; Day, Graham, Read, & Kl, 2007), and stimulating immunopathology (Graham, Allen, & Read, 2005). Yet, despite immune defense against parasites being subject to strong selection pressures from individual morbidity and mortality, heterogeneous immune expression and variation in susceptibility are still observed between hosts (Hayward, Nussey et al., 2014; Zuk & Stoehr, 2002).

According to life‐history theory, heterogeneity in immune defense and infection level can be accounted for in part by competitive resource‐allocation occurring between immune responses and other host life‐history traits (Sheldon & Verhulst, 1996; Stearns, 1992; Zuk & Stoehr, 2002). Consequently, studies suggest that sex differences in infection exist because of differing optimal fitness strategies, mediated by competitive trade‐offs between reproduction and self‐maintenance, with males favoring paternity and females longevity (Folstad & Karter, 1992; Hamilton & Zuk, 1982; Hayward, 2013; Mills et al., 2009; Zahavi, 1975). Within a host population, juveniles, and juvenile males in particular (Clutton‐Brock & Pemberton, 2004; Wilson, Grenfell, Pilkington, Boyd, & Gulland, 2004), often experience the heaviest parasite loads (Brzeski et al., 2015; Jones, Crawley, Pilkington, & Pemberton, 2005). This may be due in part to the fact that immune function is not yet fully developed in maturing individuals (Simon, Hollander, & Mcmichael, 2015), as well as potential trade‐offs arising between growth and immunity (Medley, 2002; Tschirren & Richner, 2006). Additionally, in dimorphic species, males tend to grow at a faster rate than females (Young & Bennett, 2013) and require more energy, further limiting the allocation of resources toward immune function. Finally, the processes of gestation and parental care are extremely resource‐demanding in many species (Speakman, 2008). Parasitism can seriously impair a host's physical condition, which may consequently affect energetically costly reproduction. Female mammals bear costs of reproduction, both during pregnancy and postparturition through postnatal care (e.g. lactation). While females typically harbor lower parasite loads than males, those investing heavily in reproduction may experience depressed immune function and higher parasite loads (Turner, Versfeld, Kilian, & Getz, 2012).

Although many studies have identified variation in parasite load within populations, an outstanding question is whether those host demographics, previously identified as susceptible to infection, are also found to have a higher risk of mortality from parasitic causes. Given that certain individuals suffer from increased parasitism and parasites are thus aggregated within host populations (Shaw & Dobson, 1995), it is important to determine patterns of cause‐specific mortality to identify those most at risk. There is empirical evidence that in certain conditions, those hosts that are more exposed to parasites (LoGiudice, 2003) or that have increased parasitic loads (Craig, Jones, Pilkington, & Pemberton, 2009; Craig, Pilkington, & Pemberton, 2006; Pulkkinen & Ebert, 2004) are also at higher risk of mortality. Many studies have explored aspects of parasitism‐led mortality using analytical and theoretical methods (Anderson & Gordon, 2009; Anderson & May, 1978; Best & Hoyle, 2013; Cattet et al., 2014; May & Anderson, 1978; Miller, White, & Boots, 2007; Rousset, Thomas, Meeûs, & Renaud, 1996; Wilber, Weinstein, & Briggs, 2016). Additionally, general associations between mortality and parasitism have been studied experimentally in model systems such as fruit flies, *Drosophila* spp. Izhar, Routtu, & Ben‐ami, 2015; Jaenike & Benway, 1995; Polak & Starmer, 2015) and small or shorter lived vertebrates such as Arctic charr *Salvelinus alpinus* (Knudsen, Amundsen, & Klemetsen, 2002) and European bitterling *Rhodeus amarus* (Francová & Ondračková, 2013). However, cause‐specific mortality patterns for distinct host demographics are poorly understood in natural systems (Ricklefs, 2008) and there are few studies that focus on determining demographics susceptible to particular mortality causes. Specifically, studies on parasite‐specific mortality patterns are particularly lacking for long‐lived hosts (Watson, 2013). This is understandably challenging in wild systems, where it is difficult to assign reliable causes of death to known cases of mortality. However, understanding parasite‐related mortality is important, as parasites can potentially act as both proximate and ultimate causes of death by exerting both direct and indirect health effects on hosts.

Parasite‐driven mortality risk may vary with the sex and age of hosts, but without evidence obtained from natural populations, we cannot fully understand the selection pressures exerted by parasitism. Here, we investigate the drivers of parasite‐associated mortality in Asian elephants (*Elephas maximus*) using a multigenerational database collected from a semi‐captive elephant population working in timber camps across Myanmar (formerly Burma). Our study includes longitudinal data for over 4,000 elephant records, spanning over 75 years and including information on health, lifespan, and female reproductive history for individuals varying in their age at death from under one day to over 66 years. A very significant advantage of this study population is the rare availability of cause‐specific mortality data, with trained veterinarians diagnosing proximate cause of death at the time of death or through a broad postmortem. Our semi‐captive population thus provides an unparalleled opportunity to determine the mechanisms driving mortality (Ricklefs, 2008) in a large‐bodied, long‐lived vertebrate. In this study, we use detailed causes of death to explore patterns of parasite‐induced mortality among elephants, focusing on variation in relation to individual age, sex, and reproductive history.

## 2 MATERIALS AND METHODS

### 2.1 Study population

Asian elephants are classified as endangered on the International Union for Conservation of Nature Red List (IUCN 2016) and are declining across most of their range, which is severely fragmented over Southeast Asia (IUCN 2016; Sukumar, 2006). Myanmar houses the world's largest population of captive Asian elephants (>5,000), with government‐owned working elephants, employed and centrally managed by the Myanma Timber Enterprise (MTE), contributing to over half of this number (Mar, 2002). MTE elephants have been used for draft power and transportation, each working under the authority of a human supervisor (“mahouts”) in logging camps across the country for more than a century (Toke Gale, 1971). Historically, around half or more of the working population has consisted of captive‐born individuals, whereas the remaining have been recruited by capturing wild elephants. Captive‐born calves remain with their biological mothers and allomothers (other females who participate in caretaking of a calf), being classed as “calves at heel”, until the ages of 4–5 years after which they undergo training, prior to entering the workforce. Workloads are designated by strict regulations and set predominantly by age categories (“training calves” aged 5–17 years, “working adults” aged >17–53 years, and “retired adults” of over 53 years), and to reflect seasonal changes in environment (Mar, 2007). All elephants are rested during the hottest months of the year (mid‐February–mid‐June), and they then work throughout the remaining calendar months with set maximum hours of work per day (Mar, 2007). The exception are reproductive females, which are rested for approximately half of their gestation period and the first year following parturition, and thereafter given light work for the subsequent nursing period (Mar, 2007).

The working elephants are assigned a unique identification number which is marked on their haunches, enabling recognition of every individual in the population. Data on individual life‐history, health, and maternal lineage for each elephant are recorded in an individual logbook. This includes elephant identification number (ID), origin (captive‐born or wild‐caught), date of birth (estimated for wild‐captured elephants), sex, birth region, mother ID, treatment schedules, pregnancies and calving events, and dates and causes of death or exit from the population. The elephants exhibit significant variation in parasite infection (Lynsdale et al., 2015), and infection has been a concern for the health of working elephants in Myanmar for over a century (Evans, 1910). Yet despite a substantial number of studies investigating the drivers of mortality risk in this population (Crawley et al., 2017; Hayward, Mar, Lahdenperä, & Lummaa, 2014; Lahdenperä, Mar, & Lummaa, 2016; Mar, 2007; Mumby, Courtiol, Mar, & Lummaa, 2013a), it is unknown how parasitism contributes to mortality and whether certain host traits are associated with risk of parasite‐induced mortality.

The working elephant reproduction (including calving and nursing) is unsupervised, and the elephants' diet has traditionally not been supplemented. As the elephants exhibit natural breeding and foraging behavior, their physical condition and many aspects of their life history including reproductive and survival rates are highly comparable to those observed in natural populations, rather than those of elephants in fully captive populations (Clubb et al., 2008; Hayward, Mar et al., 2014). Antiparasitic treatments such as ivermectin have been implemented relatively recently (1990s; personal correspondence with Dr Win Htut) and have since been used sporadically. Additionally, due to the close proximity between the semi‐captive population and the surrounding free‐living population and the freedom of both populations to interact at night, transmission of pathogens can occur between the two populations. Thus, it is likely that the semi‐captive timber elephants are host to similar parasites as those found in wild hosts and therefore afford insights into parasite‐driven mortality patterns in free‐living herds.

### 2.2 Data selection

From a digitized database of logbook information for over 8,000 elephant records (Lahdenperä, Mar, & Lummaa, 2014; Mar, 2002; Mumby et al., 2015; Robinson, Mar, & Lummaa, 2012), we restricted our dataset to individuals with a known ID number, sex, exact date of death or censorship, and a known cause of death. This resulted in a primary dataset of 4,242 individual elephant records, spanning from 1941 (earliest date of entry) to 2016 (latest date of death or censorship), with 2,476 censored individuals and 1,766 recorded elephant deaths. Censorship is the last known date an elephant was observed alive within the population (*n* = 2,329), or the date of exit from the population by transfer, sale, or loss (*n* = 147), rather than death. Both captive‐born (*n* = 2,914) and wild‐caught (*n* = 1,328) elephants were included in the dataset, with captive‐born individuals having an exact recorded date of birth and therefore an exact age at death. For wild‐caught elephants where age is estimated upon capture, we restricted our data to only those caught at an estimated age of 16 years or younger. For both sexes, 90%–95% of height growth is completed by age 15, coinciding with sexual maturity at 15–16 years (Mumby et al., 2015). As estimated age at capture is partially based on body size, alongside a number of other physical characteristics including skin and body condition, skin pigmentation, and tail hair density (Arivazhagan & Sukumar, 2008), estimated ages are believed to be more accurate while growth is still ongoing. The elephants are subject to routine checks by trained veterinarians throughout their lifetime, who closely monitor changes in their body condition, health, and specific illness on a monthly basis, aiding the correct diagnosis of mortality. For deceased elephants, known proximate causes of death (e.g., “worms,” “liver fluke”) were diagnosed at the time of death by vets, from previous diagnosis of illnesses and postmortem. These raw, proximate causes, as recorded by the employed vet at the time, were then categorized as “Accidental” (22.5% of all deaths), “Acute or Chronic Illness” (32.8%), “Anthropogenic” (3.2%), “Conflict” (6.6%), “Ectoparasitic” (2.0%), “Endoparasitic” (8.0%), “Reproductive” (10.6%), “Senescence” (3.3%), “Stochastic” (0.8%), “Stress” (2.8%), or “Unknown” (7.4%). Deaths which were “unknown” were recorded for elephant deaths where no cause was given in the logbook entries. Conflict, for example, can refer to known aggression from other animals as well as from conspecifics, “Stress” to mortality from taming attempts, and “Senescence” to deaths listed by veterinarians as “old age” in logbook entries, without more specific diagnosis (associated with general deterioration for elephants with a lifespan of 50–67 years). As such, our data are comparable to studies investigating historic causes of death in human populations, for example, from multigenerational parish records that are commonly used in epidemiological research (Hayward, Rickard, & Lummaa, 2013; Hayward, Rigby, & Lummaa, 2016), with the exception that in our study the causes of death were determined by trained health workers with long history of previous health of the animal in question. The MTE elephant population has not been subject to culling (Mar, 2002). As such, deaths classed as anthropogenic (56 in total) were those caused by civil unrest, insurgence, or poaching for ivory only.

Across vertebrates, while debilitating effects of parasites are commonly observed, identification of parasitism as a direct cause of death is rare and limited to cases where large, overwhelming parasite burdens lead to mass tissue damage and organ failure (Fowler & Mikota, 2006). However, such debilitating effects may ultimately also lead to host mortality. For the working timber elephant population, both endoparasitic and ectoparasitic infections are linked to clinical conditions listed as causes of death in the database, for example, “anorexia” (Fowler & Mikota, 2006). Therefore, known deaths were classed as being either (1) unrelated to parasitism (*n* = 1,030) or (2) potentially parasite‐associated (*n* = 605). The latter included instances of parasites being the listed (proximate) cause of death (e.g., “worms”) where deaths are assumed to be due to overwhelming evidence of extremely heavy infestation, diagnosed from indicative physical signs and by actual observation of parasites. Such deaths were mainly due to endoparasitic (80.1%) causes. All documented ectoparasitic deaths were described as “bots,” which are classed as ectoparasites as they are mainly free‐living, despite their main pathogenic effects deriving from subcutaneous development within the host. Heavy bot infections can have similar pathogenic effects to those of internal parasites including gastritis and damage to the gastrointestinal tract (Fowler & Mikota, 2006), and therefore are likely to contribute to host morbidity and death in a similar manner to internal parasites. Potentially parasite‐associated deaths also included cases where the proximate cause determined at the time of death was a well‐known symptom or commonly observed consequence of parasitism, rather than parasitism being listed as a direct cause. These causes denoted exhaustion (“general weakness,” “heat stroke,” and “fatigue”) or digestive and gastrointestinal problems (including, but not limited to, “diarrhea,” “liver damage,” and “malnutrition”). Several smaller, undefined but potentially parasite‐associated causes included those due to general infection (e.g., “infectious disease,” “unknown disease”), “infections” of organs where endoparasites are commonly found, and “sudden” deaths with no apparent proximate, instigating factors. Deaths with an unknown cause (which were listed as such in logbooks, *n* = 131) were excluded from analysis.

### 2.3 Statistical analysis

#### 2.3.1 Elephant age and sex

We carried out all analyses using *R* 3.1.2 (R Core Development Team 2015) with all models run using *glmer* from the *lme4* package (version 1.1‐7; Bates, Maechler, Bolker, & Walker, 2014). Predicted probabilities were calculated using *predictInterval* from the *merTools* package (version 0.1.0; Knowles & Frederick, 2015). We used two separate subsets: one for non‐parasitic and one for potentially parasite‐associated deaths. Both subsets included the same records for censored elephants (without a recorded death event and known to be alive up to a given date). For each individual, we predicted probability of cause‐specific elephant mortality per year of life spent in the working population, that is, from birth or capture up to year of death or censorship. We used a discrete time‐event analysis with each year of life spent in the population classed as a separate time unit and entered as single observations following Allison (1982) and Mumby, Courtiol, Mar, and Lummaa (2013b). For each elephant‐year, mortality was classed as either “0” if spent alive or “1” for a year in which a death event occurred. For captive‐born elephants, this included every year of life from birth until age at departure from the population, whereas for wild‐caught elephants entries commenced at the year of capture (as estimated age at capture) until year of exit. This resulted in a dataset consisting of 78,691 observations, derived from the total 4,242 elephant individuals.

We analyzed mortality risk using a logistic mixed‐effects model framework with binomial error structure and logit link function. We ran a series of separate models: one for each of the non‐parasitic or potentially parasite‐associated data (vs. censored individual) subsets. We also ran models comparing non‐parasitic to potentially parasite‐associated deaths for inclusivity; however, as these results were not a main focus of this study, they are not discussed but are instead only included in the Table S1. Our approach permits individual elephants to be accounted for across differing ages of entry (either varying capture ages or from birth) and exit, using elephant‐years as discrete time intervals. We therefore include individuals only when they are present in the population and adjust for the fact that we do not include wild‐caught elephants, which may be missed from the sample due to dying at a very young age before possible capture, from birth. We also avoid deaths being disproportionately represented at extremely young or old ages, due to fewer censored elephants surviving to old age and due to wild‐caught elephants not accounted for from birth but instead being represented at older ages in the population following capture. To first determine the association between age and mortality risk, we compared models with age (calculated from precise or estimated date of birth until date of death or censorship) as continuous linear, quadratic, and cubic terms using a likelihood ratio test (LRT). The best fitting model structure (with the highest hierarchal level of significance) was included in base models.

Fixed effects incorporated into the base model framework included sex as a two‐level categorical factor (“female” or “male”) and origin, also a two‐level categorical factor of “wild‐caught” or “captive‐born.” We account for the introduction of anthelmintic treatment in our analysis, although we consider this unlikely to have a large effect: Dates for deaths known to be caused by endoparasitic infection range from 1965 to 2007 even though the use of ivermectin was implemented in the 1990s. We included treatment as a fixed binary term, classing a death or censorship occurring before treatment was introduced (pre‐1990) as “0” or “1” if occurring after (post‐1990). To control for the effects of capture on mortality, time since capture was included as a continuous covariate, but only as an interaction term with origin (i.e., no main effect of time since capture was included in the models). For wild‐caught elephants, this was coded as “0” at the year of capture and increasing cumulatively hereafter (e.g., “1” the year following capture, “2” the year after). As captive‐born elephants were continually set to “0,” this approach allowed us to adjust for time since capture only for the wild‐caught animals in our analysis. We also included an interaction term between age (as a linear continuous variable) and sex. This accounted for sex‐specific changes in behavior and physiology which may affect survival, for example deaths attributed to musth, a testosterone fueled peak in aggression in reproductive males (Mar, 2007). For each known subset (non‐parasitic, potentially parasite‐associated), we tested the interaction term by removing it from the base model and then comparing to the base model using an LRT. Three random terms were incorporated into the model structure; “birth region” across different states of Myanmar (categorical with 13 levels in total), “birth decade” (10‐level categorical factor based on estimated or actual date of birth), and identification number (“ID”) of each elephant, in order to account for repeated measures across regions, cohorts, and individuals. By including these terms, we controlled for between‐cohort variation in mortality and regional variation in environment such as temperature and rainfall which are known to affect mortality within the working elephant population. Through controlling for geographical (birth region) and longitudinal (birth decade) variation, specific to individual elephants (with ID included as a random term), our analysis also captured changes in population density through space and time.

#### 2.3.2 Female elephant reproduction

To investigate the effect of female reproduction on parasite‐driven mortality risk, we further restricted our subsets to females of 8 years of age and above, which was the earliest age of reproduction in our sample. This resulted in a total of 1,657 females of reproductive age, comprising 796 individuals that had already produced offspring and 861 nonreproducers before death or censorship. Of the 796 reproducers, 137 died from non‐parasitic causes, 75 from potentially parasite‐associated causes, and 25 of unknown cause.

We investigated the relationship between mortality and female reproduction using two separate model approaches for both mortality subsets. As before, we ran models comparing the two different death causes, but only present results in the Tables S2 and S3. First, we analyzed the relationship between long‐term reproductive output and mortality with a two‐level categorical variable (reproducer vs nonreproducer) describing whether females of reproductive age had produced any offspring within the time spent in the population. Second, we investigated the effect of reproduction which occurred within close proximity to death. Of the total 1,657 individuals, 400 females reproduced within 5 years of exit from the population, including 314 censored and 86 dead individuals. These deaths included 40 due to non‐parasitic causes, 33 classed as potentially parasite‐associated, and 13 of unknown cause. We thus included a two‐level categorical fixed factor to describe whether females had produced offspring within a maximum 5 year period (or within the total time spent within the population if this was less than 5 years—“short‐term” reproduction) preceding individual date of death or censorship (“yes” or “no”). Some elephants (*n* = 76) were present in the population for less than 5 years, but were included in the analysis as their addition did not substantially affect the results for our primary terms of interest (age and reproduction). Five years was selected as a measure of recent female reproductive investment due to the average length of gestation for Asian elephants being 22 months, with weaning known to occur approximately 2–3 years after birth (Fowler & Mikota, 2006) and mean birth intervals in this and other elephant populations being around 5 years (Mar, Lahdenperä, & Lummaa, 2012). Additionally, reproduction is known to increase overall mortality in the working elephants in the 5 years following parturition (Robinson et al., 2012). In both analyses, base models were constructed as previously described but excluding a term for sex and instead including fixed terms for either long‐ or short‐term reproduction.

## 3 RESULTS

### 3.1 Cause‐specific mortality

**Figure 1:**
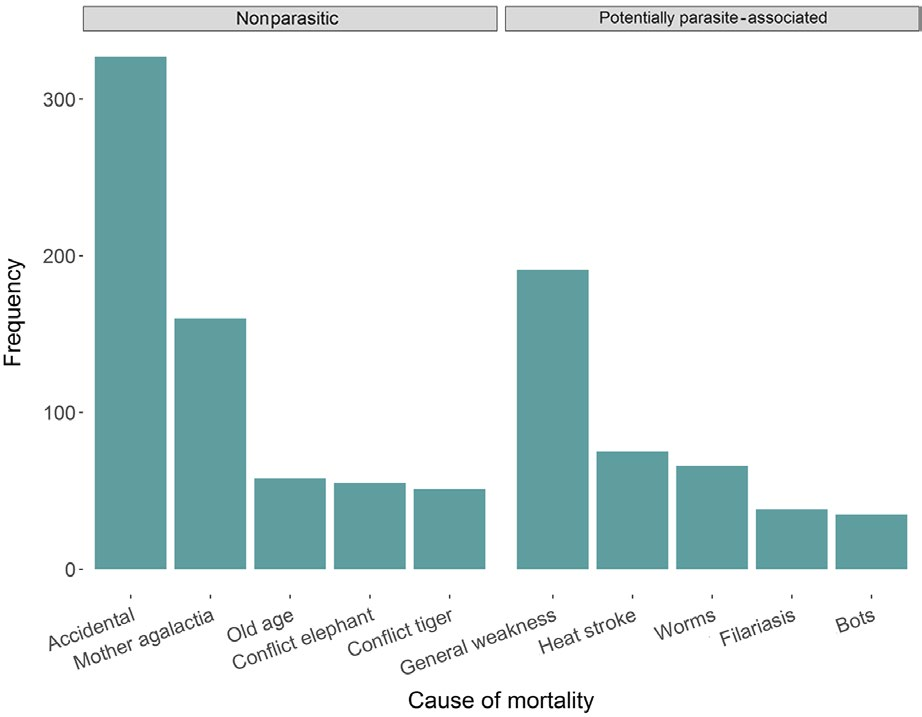
Most common documented causes of death for both potentially parasite‐associated and non‐parasitic causes from the final dataset by frequency (total no. of recorded elephant deaths, *n* = 1,766). “Mother agalactia” refers to deaths caused by a lack of milk production by nursing mothers, and “Conflict elephant” refers to deaths caused by aggression from other conspecifics, whereas “Conflict tiger” refers to those caused by aggression from tigers. Of all recorded deaths, only one other cause (taming stress, *n* = 50) had a higher occurrence than the deaths described in Figure 1. Causes were adapted from raw entries in logbooks

Lifespan of elephants ranged between one day and over 66 years, with the average lifespan being 32 years. Overall, parasites were directly attributed to causing approx. 10% of all recorded deaths in our final dataset (*n* = 176/1,766), with the most common cause within this category being listed as endoparasitic worms. Additionally, we found “worms” to be among the five most frequent causes of potentially parasite‐associated mortality (Figure 1). Deaths classed as potentially linked to parasitism accounted for a total of 34.3% of mortalities (*n* = 605), including those described as due to “general weakness” (*n* = 191) and “heat stroke” (*n* = 75).

### 3.2 Potentially parasite‐associated mortality

**Figure 2:**
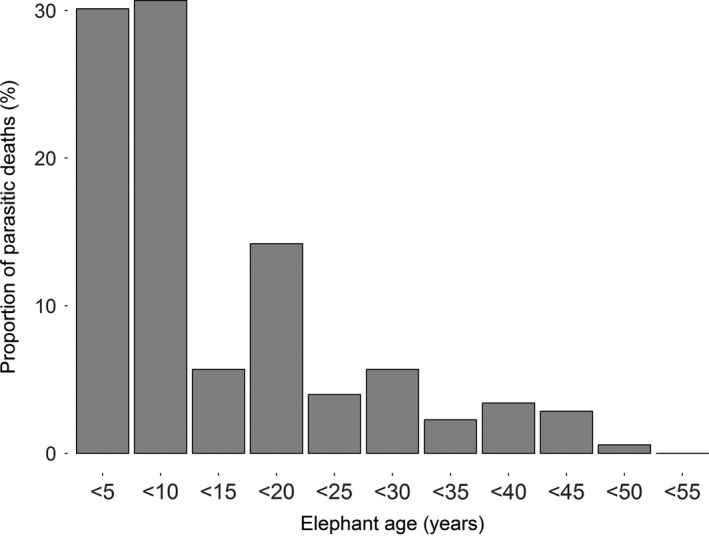
Percentage of deaths directly attributed to parasitism as raw logbook entries (e.g., as “worms”) across elephant ages (176 records of parasitic deaths of 1,766 total deaths: One death falling outside the displayed age range is omitted)

**Figure 3:**
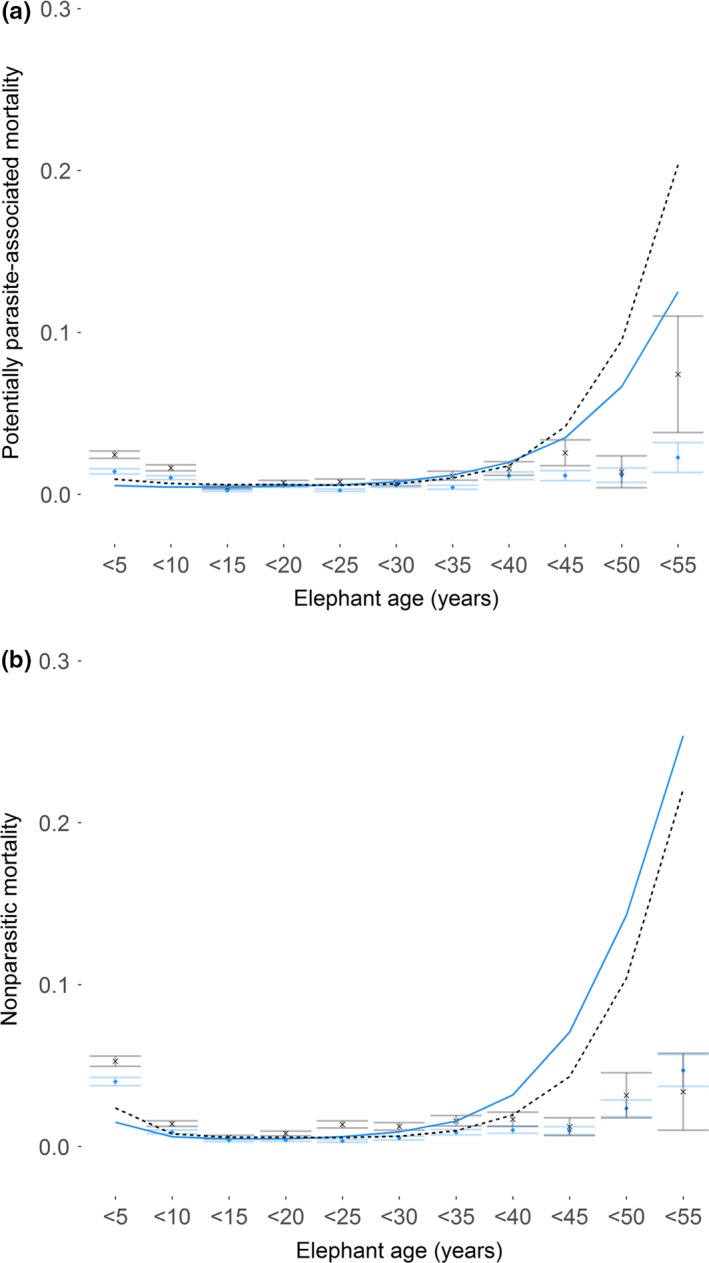
Raw calculated rate (points) and model predicted (lines) elephant mortality due to (a) potentially parasite‐associated (*n* = 605 dead and *n* = 2,476 censored individuals) and (b) non‐parasitic (*n* = 1,030 dead and *n* = 2,476 censored) causes across different elephant ages and for different sexes (females are represented by blue lines and points and males by gray dotted lines and points). Raw mortality was calculated as number of death events (total “1”) per age‐year/total number of entries per age‐year (total “0” + total “1”). Plotted points are averaged mortality within 5‐year bins with standard error bars. Plotted data are restricted to elephants aged 55 years and under

Over 60% of recorded deaths where parasites were a direct cause occurred in elephants younger than 10 years of age (Figure 2), with mortality highest for calves aged between 5 and 10 years than any other age group. We found the probability of dying of potentially parasite‐related causes varied significantly as a quadratic function of age in elephants (χ^2^ = 3.497, *p* = .061 for the cubic term, χ^2^ = 104.930, *p* < .001 for quadratic, and χ^2^ = 108.780, *p* < .001 for linear), with the youngest and oldest elephants at the extremes of lifespan being more likely to die of potentially parasite‐related causes than adults at prime ages (Figure 3a). Risk differed significantly between sexes (χ^2^ = 40.331, *p* < .001), with males having significantly higher risk of parasite‐induced mortality than females (model estimate ±*SE* = 0.578 ± 0.090, *z* = 6.451, *p* < .001; see Table S1). There was no significant interaction between age and sex (χ^2^ = 0.040, *p* = .8410), suggesting that sex differences in mortality risk were maintained across different elephant ages. Finally, we found no significant difference in potentially parasite‐associated mortality between elephants of different origin, with wild‐caught elephants being no more likely to die of such causes than those born into the working population (estimate ± *SE* = −0.044 ± 0.151, *z* = 0.293, *p* = .770).

### 3.3 Nonparasitic mortality

The probability of dying of nonparasitic causes varied significantly with age as a cubic term (χ^2^ = 5.593, *p* = .018 for the cubic age term, χ^2^ = 217.97, *p* < .001 for the quadratic, and χ^2^ = 76.505, *p* < .001 for the linear age term). As with the results for potentially parasite‐associated deaths, we found that the youngest and oldest elephants (i.e., those at the extremes of lifespan) were more likely to die of nonparasitic causes than adults at prime age (Figure 3b). We found a significant sex difference with regard to nonparasitic mortality (χ^2^ = 28.454, *p* < .001), with males also having a significantly higher risk of non‐parasitic mortality than females (model estimate ±*SE* = 0.398 ± 0.075, *z* = 5.207, *p* < .001; see Table S1). An interaction between linear age and sex was found to be non‐significant (χ^2^ = 2.646, *p* = .104); however, males aged approximately 30–55 years had a lower predicted probability of death from non‐parasitic causes than females. Additionally, opposite to the trend observed for potentially parasite‐associated deaths, we found a difference in non‐parasitic mortality risk between elephants of different origin, with wild‐caught elephants having significantly lower risk than captive‐born elephants of dying from non‐parasitic causes (estimate ±*SE* = −0.854 ± 0.165, *z* = −5.172, *p* < .001).

### 3.4 Female reproduction

**Figure 4:**
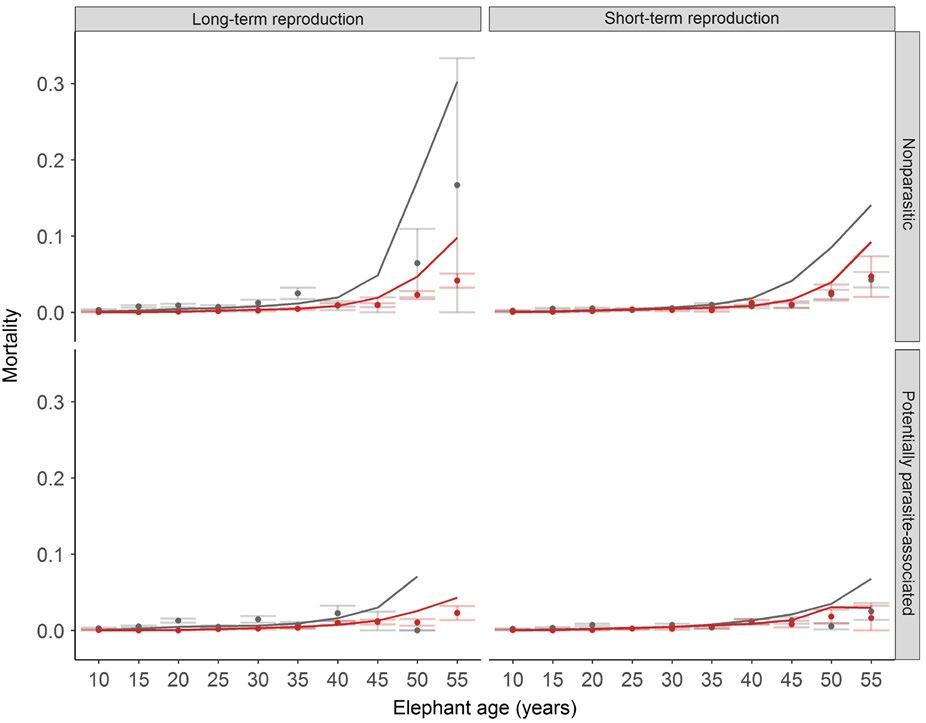
Raw (points) and model predicted (lines) elephant mortality according to reproductive effort in the short and long term. Non‐reproducers are in gray and reproducers in red. Top panels show non‐parasitic mortality for reproduction in the long term (total time spent in the population, *n* = 220 dead and *n* = 1,243 censored individuals) and short term (within <5 years of exit, totals as with long term) across different elephant ages. Bottom panels show mortality due to potentially parasite‐associated causes for long‐term and short‐term (both *n* = 148 dead and *n* = 1,243 censored) reproduction. Raw mortality was calculated as number of death events (total “1”) per age‐year/total number of entries per age‐year (total “0” + total “1”) with standard error bars. Plotted data are restricted to elephants aged 55 years and under. For clarity, bottom panels are also plotted on a smaller scale in Fig. S1.

48.0% of females in our sample had reproduced, with 29.8% of reproducers within our sample dying. We found that female reproducers had a significantly lower probability of dying from potentially parasite‐associated causes in both the long term (comparing parasite‐associated mortality of those females who did vs. did not reproduce within the total time they spent within the population, model estimate ±*SE* = −1.636 ± 0.247, *z* = −6.617, *p* < .001; see Figure 4 and Table S2) and short term (those dying within a maximum of 5 years from reproduction, model estimate ±*SE* = −0.789 ± 0.217, *z* = −3.637, *p* < .001; see Figure 4 and Table S3). Similar results were observed for non‐parasitic mortality, with non‐reproducers again being significantly more at risk than reproducing females over both time scales (long‐term model estimate ±*SE* = −1.776 ± 0.271, *z* = −6.548, *p* < .001; Figure 4, Table S2; short‐term model estimate ±*SE* = −0.724 ± 0.194, *z* = −3.742, *p* < .001; Figure 4, Table S3).

## 4 DISCUSSION

Using a multigenerational database, we investigated the risk of potentially parasite‐associated mortality with differences in host age, sex, and reproductive status in a semi‐captive population of Asian elephants. We found that male elephants, extremely young (<5 years) and older (>50 years) elephants, and non‐reproducing females were more vulnerable to potentially parasite‐associated mortality than females, adults at prime age, and mothers. Individual data on parasite load, corresponding to specific causes of death, were not available for our study and so we did not draw associations between level of infection and mortality risk in the elephants. However, we found elevated potentially parasite‐related mortality risk in certain demographic groups, namely males, juveniles, and elderly adults, which previous studies have identified as being most susceptible to infection and high parasite burdens.

Juvenile hosts typically experience higher parasitism, possibly as they take time to develop an acquired immunity to infection (Sol, Jovani, & Torres, 2003). Reduced immune memory may also lead to increased parasite‐driven mortality risk through increased parasite loads and reduced capability to respond efficiently to initial infection. Increased general calf mortality within the first 5 years of life has been previously reported within the Myanmar elephant population (Lahdenperä, Mar, & Lummaa, 2015; Mar et al., 2012). However, our study is one of the few to explore parasitism‐related mortality patterns in an extremely long‐lived mammal, showing that those at the extremes of lifespan (youngest calves and oldest adults) are most at risk. The drivers underlying potentially parasite‐associated mortality in elephants may be different for individuals lying at opposite ends of elephant lifespan, for example, through lack of acquired immunity or immunosenescence (Doolan, Dobaño, & Baird, 2009). Specifically, youngest elephants may suffer from condition‐dependent mortality (Hämäläinen et al., 2014), with parasites facilitating the removal of low‐quality hosts within the population. Older elephants may have accumulated experience of repeated infections throughout their lifetime and may consequently suffer increased morbidity and mortality due to chronic exposure (Castle, Uyemura, Fulop, & Makinodan, 2007) rather than as a result of later life parasitism. In such instances, repeated exposure itself may be a cause of immunosenescence. We observed similar patterns of mortality risk for elephants dying of both potentially parasite‐associated and non‐parasitic causes. Interestingly, parasite‐associated mortality risk in older elephants differs to disease‐driven mortality trends in human (*Homo sapiens*) populations (Lozano et al., 2012). In humans, mortality due to disease peaks in younger children, similarly to our elevated mortality risk in calves. However, human mortality linked to infectious disease then rises again, and declines after approx. 39 years of age until the end of lifespan. Advanced medical care may be more commonly available to humans, especially those living in developed countries. As such, differences between mortality trends may instead correspond to conditions pertaining to chronic illness, cancer, or to human‐associated lifestyle factors, for example, high levels of cardiac‐related mortality, as observed in human populations (Lozano et al., 2012). Besides age‐related inability to respond efficiently to infections, an additional factor increasing mortality, specifically among calves aged four or five years in our study population, is their taming and training to working life. At four or five years of age, captive‐born elephants are weaned and undergo taming, which is considered stressful (Toke Gale, 1971). For wild‐caught elephants, the process of capture and taming is highly stressful (Mar, 2007). Increased stress levels have been associated with increased parasitism and infectious disease in vertebrate species (Hing, Narayan, Thompson, & Godfrey, 2014). The additional stress of taming may therefore exacerbate the effects of infection, potentially increasing mortality risk in extreme cases, for certain individuals, although further research is needed to confirm this.

Potentially parasite‐associated mortality differed significantly between sexes, with males having a higher overall risk of dying of parasites than females. Additionally, we found that males also had a significantly higher risk of dying of non‐parasitic causes than females. A male‐biased mortality is a common finding in many mammals, including within the working elephant population we studied (Mar et al., 2012; Mumby et al., 2013b). Yet, little is known about the driving forces and underlying causes of this sex‐specific disparity. Male‐biased parasitism, a common trend within vertebrates, is thought to arise due to sexual selection pressures driving resource allocation trade‐offs in favor of reproduction over longevity and immune function (Harrison, Scantlebury, & Montgomery, 2010). Endocrinological sex differences are potential mediators of this dynamic: for example, testosterone is considered to have immunosuppressive effects in vertebrates (Balenger & Zuk, 2014), which can act in synergy with behavioral or environmental factors. Hämäläinen, Raharivololona, Ravoniarimbinina, and Kraus (2015) found elevated male parasitism in mouse lemurs (*Microcebus murinus*) to be seasonal, coinciding with an annual peak of testosterone levels and increased male roaming behavior. Male Asian elephants are known to experience musth, an increase in androgens, including testosterone, resulting in increased aggression and sexual behaviors which can persist for several months (Fowler & Mikota, 2006; Somgird et al., 2016; Sukumar, 2003). Little is known of the association between musth and infection dynamics, which may be an interesting avenue for future research as such hormonal changes could potentially have immunosuppressive effects and hence affect mortality risk.

In females, pregnancy and post‐reproductive care are highly energetically costly, leading to compromises in immune function through life‐history trade‐offs. Such resource competitive exchanges have been observed between reproductive investment and immune function in domesticated sheep (*Ovis aries*; Beasley, Kahn, & Windon, 2010; Houdijk, 2008). Similarly, in spotted hyenas (*Crocuta crocuta*), lactating females and mothers nursing twins had significantly higher parasite loads than their non‐lactating or single‐offspring mother counterparts (East et al., 2015). If links between increased infection and higher parasite‐linked mortality exist for mothers, mortality risk of this cohort may in theory be further exaggerated by increased infection during the reproductive period, particularly if such dynamics occur during resource‐limiting environmental conditions (such as drought). To our knowledge, no associations have been established between survival, reproduction, and infection in many wild or semiwild systems, comparable to those for Asian elephant mothers in our study population. Previous studies have shown that heavy investment in fecundity prior to the peak reproductive age of 19 significantly impairs subsequent overall survival in female Asian elephants (Hayward, Mar et al., 2014), but the specific causes have remained elusive. In contrast to our prediction, we found that female reproducers had a significantly lower mortality risk of potentially parasite‐associated causes than nonreproducers. This held true when investigating the effects of reproduction on parasite‐related mortality both across a long‐term observable window (throughout time spent in the population) and in the short term (proximate to departure from the population, within a maximum of 5 years from exit). An alternative explanation may be that females in poorer health favor survival over fecundity, and delay reproduction in light of heavy parasite loads, instead allocating resources to immune function. However, further empirical studies linking parasite burdens, immune responses, and reproductive status would be needed to support this line of thought.

While parasites have been implicated in the regulation of certain populations of red grouse *Lagopus lagopus scotica* (Hudson, Dobson, & Newborn, 1998), Arctic charr (Knudsen et al., 2002), and Svalbard reindeer *Rangifer tarandus platyrhynchus* (Albon et al., 2002), studies describing the frequency of parasite‐induced mortality and identifying the individuals within populations with the highest risk of parasite‐induced mortality are exceptionally rare. In addition, while studies have shown that infection intensities within wild populations are consistently increased in certain groups, for example, juveniles (Brzeski et al., 2015) and males (Craig et al., 2006; Harrison et al., 2010; Klein, 2004), links between these groups and elevated mortality risk have rarely been made. Arguably in many wild vertebrate populations, individual mortality is likely to be due to a synergy of factors, for example, interactions between infection and weight loss, hormone differences, and poor diet. For example, in the Soay sheep population of St. Kilda, dead individuals after winter population crashes have been found harboring large parasite burdens and with signs of heavy parasite‐mediated damage, but with cause of death thought to be a culmination of low food abundance and infection (Hayward et al., 2011). Considering parasitism is well documented within our semicaptive elephant population (Lynsdale et al., 2015), such synergies could arise between infection and other factors, for example, malnutrition. Thus, despite not being listed as parasitism directly, raw causes of death that were symptomatic of infection, for example, gastrointestinal problems, were therefore included as potentially parasite‐related. We stress that although in our study we used a stringent classification system to categorize causes of death as either “non‐parasitic” or “potentially parasite‐associated,” a large degree of uncertainty is unavoidable in disentangling such causes. Nonetheless, our results provide a baseline in understanding the effects of parasitism on general mortality trends within the elephant population. As little data have existed with this focus thus far, they are of importance for developing better analytical and theoretical models for understanding parasite‐driven mortality.

In conclusion, we found variation in parasite‐linked mortality within certain demographic groups, which are commonly described in the literature as susceptible in vertebrate populations. Increased parasite‐related mortality in calves and males has important implications for captive management of Asian elephants, as well as aiding targeted conservation of wild populations. Conversely, we found mothers to be at lower risk of potentially parasite‐associated mortality than non‐reproductive females. A potential avenue of further research using corresponding data on parasite loads would be to investigate whether females suffering higher levels of infection delay reproduction, favoring survival when facing increased parasitism. Longitudinal studies are imperative for comprehensive understanding of life‐history processes in wild animal populations (Nussey, Coulson, Festa‐Bianchet, & Gaillard, 2008). Long‐term monitoring of host infection and health may help establish pathogenic threshold burdens for hosts and tease apart temporal or environmental interactions with infection dynamics. However, such studies are understandably difficult in extremely long‐lived host species due to ongoing financial and logistic requirements. For long‐lived hosts, investigating cause‐specific mortality, as we have done, and establishing phenotypic differences in incidences of parasite‐caused deaths may be a more realistic examination of parasitic mortality effects. Prolonged in situ measurement of susceptibility, such as establishing parasite loads of individual hosts and comparative rates of change in health, body condition, and nutrition parameters, coupled with findings in host‐specific mortality differences, may allow for an extensive picture of between‐individual variation of parasitic susceptibility in host populations.

## DATA ACCESSIBILITY

Data will be made available in Dryad upon acceptance of the manuscript.

## CONFLICT OF INTEREST

None declared.

## AUTHORS' CONTRIBUTIONS

CL and VL conceived the ideas and designed methodology; KM collected the data and collated the electronic database; CL led the data analysis, with contribution from HM and AH; and CL led the writing of the manuscript. All authors contributed critically to the drafts and gave final approval for publication.
