# Supplementary figures and images for: Parasite‐associated mortality in a long‐lived mammal: Variation with host age, sex, and reproduction
Source: Ecol Evol. 2017 Nov 12;7(24):10904–15. doi: 10.1002/ece3.3559 (PMC5743535; doi:10.1002/ece3.3559)

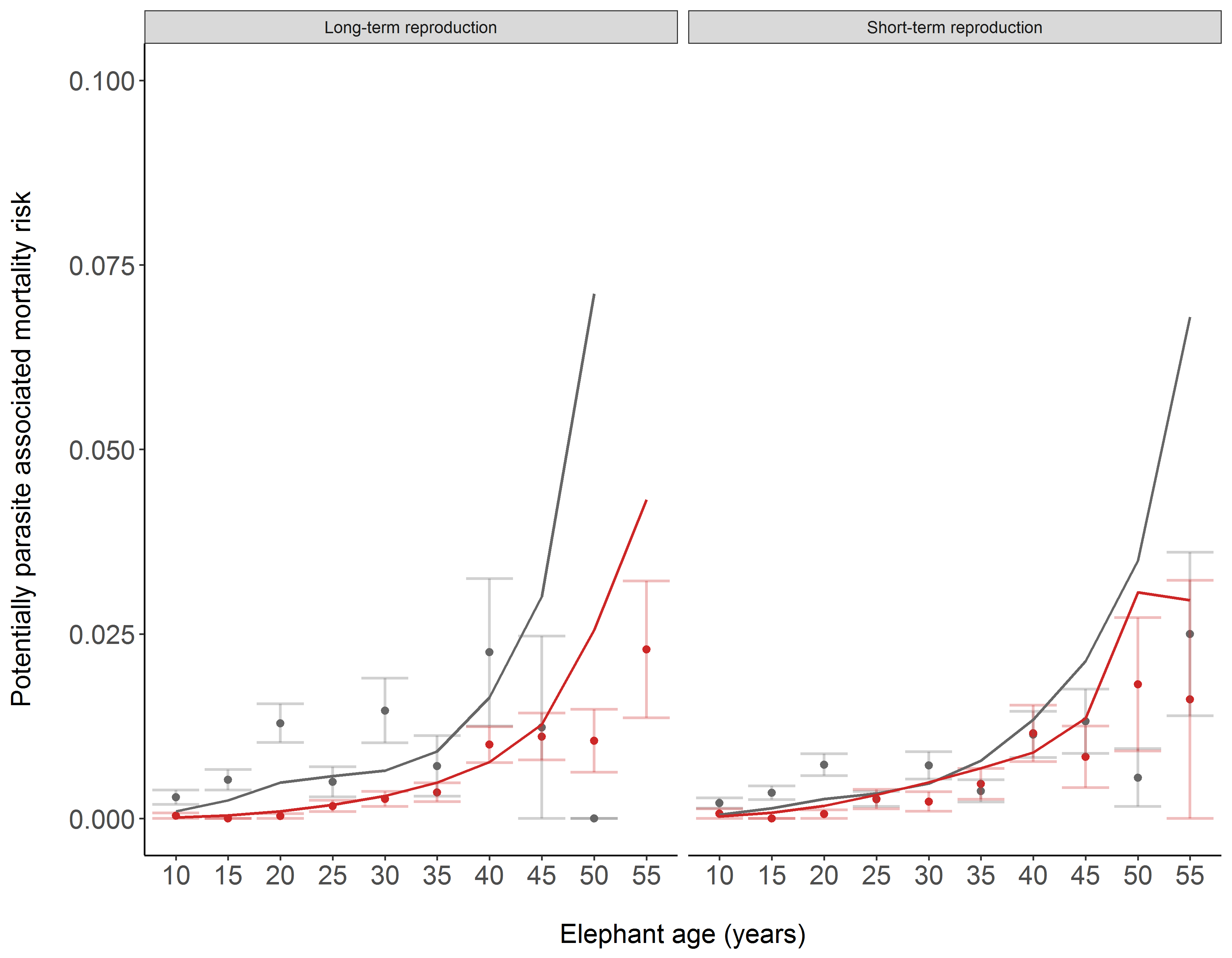

Supplement: Supplementary file 1 [file ECE3-7-10904-s001.tif]
