# Supplementary material for: Parasite‐associated mortality in a long‐lived mammal: Variation with host age, sex, and reproduction
Source: Ecol Evol. 2017 Nov 12;7(24):10904–15. doi: 10.1002/ece3.3559 (PMC5743535; doi:10.1002/ece3.3559)
Supplement: Supplementary file 2 [file ECE3-7-10904-s002.docx]

**Table S1.** Obtained fixed effects estimates from generalized linear mixed effects models with binomial error structure and logit link function. These modelled the effect of either potentially parasite associated mortality or non-parasitic mortality against censored elephants against elephant age, sex, treatment and origin. Also included are estimates for the effect of potentially parasite associated mortality vs non parasitic mortality. Estimates are expressed on the logit scale. The intercept corresponds to the predicted mortality at age 0, for female elephants, who left the population prior to the introduction of anthelmintic treatment and who were captive born. Individual elephant identification number, region and birth year were included in the models as random effects (not shown in table). Models were fitted to observations for 3081 (potentially parasite associated mortality x censored), 3506 (non-parasitic mortality x censored) and 1635 (potentially parasite associated x non-parasitic mortality) elephant records. All figures are limited to 3 decimal places.

| Model | Fixed Effect Coefficients | Estimate | Std. Error | Z Score | P Value |
| --- | --- | --- | --- | --- | --- |
| Potentially Parasite Associated Mortality x Censored | Intercept | -2.992 | 0.670 | -4.467 | <0.001 |
|  | Age (Linear) | -0.044 | 0.012 | -3.781 | <0.001 |
|  | Age (Quadratic) | 0.026 | 0.002 | 12.329 | <0.001 |
|  | Sex (M) | 0.578 | 0.090 | 6.451 | <0.001 |
|  | Treatment (Post-1990) | -3.890 | 0.163 | -23.808 | <0.001 |
|  | Origin (W) | -0.044 | 0.151 | -0.293 | 0.770 |
|  | Time_Since_Capture:Origin | 0.002 | 0.007 | 0.247 | 0.805 |
| Non Parasitic Mortality x Censored | Intercept | -1.960 | 0.713 | -2.749 | 0.006 |
|  | Age (Linear) | -0.143 | 0.019 | -7.422 | <0.001 |
|  | Age (Quadratic) | 0.053 | 0.008 | 6.947 | <0.001 |
|  | Age (Cubic) | -0.025 | 0.009 | -2.899 | 0.004 |
|  | Sex (M) | 0.398 | 0.075 | 5.207 | <0.001 |
|  | Treatment (Post-1990) | -3.874 | 0.153 | -25.396 | <0.001 |
|  | Origin (W) | -0.854 | 0.165 | -5.172 | <0.001 |
|  | Time_Since_Capture:Origin | 0.036 | 0.007 | 5.047 | <0.001 |
| Potentially Parasite Associated Mortality x Non Parasitic Mortality | Intercept | -2.525 | 1.094 | -2.308 | 0.021 |
|  | Age (Linear) | 0.036 | 0.011 | 3.359 | <0.001 |
|  | Age (Quadratic) | 0.013 | 0.001 | 8.831 | <0.001 |
|  | Sex (M) | -0.141 | 0.086 | -1.638 | 0.101 |
|  | Treatment (Post-1990) | -2.543 | 0.124 | -20.560 | <0.001 |
|  | Origin (W) | -0.377 | 0.119 | -3.165 | 0.002 |
|  | Time_Since_Capture:Origin | 0.020 | 0.006 | 3.478 | <0.001 |
|  | Age_Linear*Sex | 0.008 | 0.005 | 1.729 | 0.084 |
